# Knowledge and attitudes about vitamin D and sunlight exposure in premenopausal women living in Jeddah, and their relationship with serum vitamin D levels

**DOI:** 10.1186/s41043-021-00263-w

**Published:** 2021-08-28

**Authors:** Tahani A. Zareef, Robert T. Jackson

**Affiliations:** 1 Department of Nutrition and Food Science, University of Maryland, College Park, MD 20742 USA; 2 Department of Clinical Nutrition, College of Applied Medical Sciences, King Saud Bin Abdulaziz University for Health Sciences, Jeddah, Saudi Arabia; 3 Department of Public Health , College of Health Sciences, Saudi Electronic University, Jeddah, Saudi Arabia

**Keywords:** Vitamin D deficiency, Knowledge, Attitude, Sunlight exposure, Vitamin D sources, Premenopausal Saudi women

## Abstract

**Background:**

Saudi women are at risk of vitamin D deficiency because they are fully covered by traditional clothing and because of their indoor lifestyle. The latest national study reported that vitamin D deficiency (serum 25(OH)D < 50 nmol/L) affects 72% of young Saudi women. Because little information is available regarding knowledge on vitamin D, attitudes toward sun exposure, and the vitamin D status of premenopausal women in Jeddah, more research is necessary in order to develop effective intervention programs. The purpose of this study is to explore how the relationship between knowledge of vitamin D and attitudes about sun exposure affect the serum 25(OH)D levels in premenopausal Saudi women.

**Methods:**

This cross-sectional study included 257 women aged 20–50 years attending the primary care clinic in Jeddah, Saudi Arabia. Participants completed questionnaires about socio-demographics, dietary vitamin D intake, attitudes toward sun exposure, and were tested on their knowledge of vitamin D. Serum 25(OH)D was evaluated using chemiluminescent microparticle immunoassay.

**Results:**

Although 99% of participants had heard of vitamin D and 91% knew that sunlight exposure is a primary source of vitamin D, they also expressed the feeling of having insufficient knowledge regarding vitamin D sources. Furthermore, the majority of participants had negative attitudes toward sun exposure. High fish consumption was associated with a higher level of knowledge regarding vitamin D. The binary logistic regression indicated that low levels of knowledge about vitamin D were associated with low education levels (odds ratio = 0.397, 95% CI = [0.206, 0.765], *p* = 0.019) and with being married (odds ratio = 0.522, 95% CI = [0.281, 0.971], *p* = 0.04). In addition, spending time outside in the sun was significantly associated with increased serum 25(OH)D levels (*p* = 0.006), and the wearing of colored abaya was significantly associated with increased serum 25(OH)D levels (*p* = 0.008).

**Conclusion:**

Suboptimal vitamin D status and insufficient knowledge of vitamin D intake sources are common in premenopausal women in Jeddah. Based on this data, health professionals could provide medical intervention to the most vulnerable female patients, as well as offer clear guidelines and information to the general public.

**Supplementary Information:**

The online version contains supplementary material available at 10.1186/s41043-021-00263-w.

## Background

Vitamin D, which is an essential fat-soluble nutrient, is obtained through either synthesis in the epidermis upon exposure to ultraviolet (UVB) sunlight or by the intake of vitamin D-rich foods such as oily fish, egg yolks, veal, beef, liver, and sun-dried mushrooms. Serum concentration of 25-hydroxyvitamin D (25(OH)D) is considered the best indicator of vitamin D nutritional status, as it reflects both vitamin D produced in the skin and that acquired from the diet. While the optimal 25(OH)D level is a topic of considerable debate, the general consensus is that a serum 25(OH)D concentration < 25 nmol/L may indicate vitamin D deficiency [1–3]. As per the US Institute of Medicine’s (IOM) 2011 conclusions, a serum 25(OH)D concentration < 30 nmol/L qualifies as “deficient” and has a known impact on skeletal health, calcium malabsorption, and secondary hyperparathyroidism, and leads to rickets in children and increased bone resorption and osteomalacia in adults. Additionally, serum 25(OH)D in the range of 30–50 nmol/L may be “inadequate” in some people, while serum 25(OH)D > 50 nmol/L is “sufficient” for a majority (97.5%) of the population [4, 5]. Given those thresholds, vitamin D deficiency (< 50 nmol/L) is prevalent in many populations worldwide.

Saudi Arabia reports some of the world’s highest rates of vitamin D deficiency [6–8], with Saudi women at a particularly high risk [9, 10]. In our recent national study [11], we confirmed that vitamin D deficiency is highly prevalent (77.6%) among premenopausal Saudi women. Other Saudi studies [7, 12, 13] have highlighted concerns about Saudi women of reproductive age who had serum 25(OH)D levels of 10–45 nmol/L and were at risk of suboptimal bone health, which can lead to osteomalacia, chronic back pain, and other bone-related problems [7, 14–16]. Notably, young women are ideal candidates for the investigation because they have been found to be at risk of vitamin D deficiency and are at the domain age of developing healthy behaviors that lead to long-term health promotion and disease prevention [17].

Limited exposure to sunlight is the main contributor to low serum 25(OH)D concentrations in the Saudi Arabian population. This is the direct result of cultural practices (e.g., traditional clothing that fully covers the skin), a hot climate that drives people to seek air-conditioned comfort indoors, and the pigmentation of dark skin that reduces the endogenous production of vitamin D [6, 7, 18]. Therefore, in Saudi Arabia, it is necessary to rely on natural and fortified oral sources of vitamin D [4, 19]. Most Saudi Arabian companies fortify their dairy products, whole milk, skimmed milk, and *Laban* (buttermilk) with vitamin D to a level of 400 IU/L and 40 IU/100 g for yogurt [20]. However, our recent study [11] showed that premenopausal women in Jeddah had insufficient vitamin D intake and indicated that it was particularly low among young women, who are at greatest risk of vitamin D deficiency.

In addition to dietary intake and sun exposure, international studies [21, 22] have suggested that a lack of knowledge about nutrition and the importance of micronutrients to overall health are also contributing factors for vitamin D deficiency. Surveys in Saudi Arabia have also reported a lack of knowledge about the production and intake of vitamin D [23].

To overcome this, public health policies should include effective intervention programs that include multiple actions: fortification of food with vitamin D, vitamin D supplementation for risk groups, and the enhancement of public awareness. However, successful and effective public awareness programs currently require answers to several questions [24]. For example, what do people really know about vitamin D sources, supplementation, and food fortification? What are the prevailing attitudes regarding sun exposure and its effect on vitamin D status? Do views vary by age group? The scarcity of data relevant to these issues for the general public in Saudi Arabia, and for young women in particular, makes further studies imperative. Surveys in Saudi Arabia [25] have suggested that a high proportion of the population is unaware of vitamin D nutrition and deficiency; however, given that only a small group of students representing an educated sector of society was surveyed, these findings are limited. A general lack of knowledge points to the need for quantitative research into the level of awareness in healthy Saudi women of the importance of vitamin D intake and sun exposure and how such things impact their vitamin D status [25, 26]. Thus, the present study focuses on premenopausal women attending the primary care clinic at King Abdul-Aziz Medical City in Jeddah in an effort to examine the women overall knowledge of vitamin D, their attitudes toward sun exposure, and to explore the associations between knowledge and attitudes and their serum 25(OH)D levels.

## Methods

### Study design and participants

This cross-sectional study has been described in detail else-where [11]. Briefly, 257 premenopausal Saudi women aged from 20 to ≤ 50 years were recruited to this study. The participants were selected from attendees of the primary care clinic at King Abdul-Aziz Medical City in Jeddah, Saudi Arabia using systematic sampling. The sampling frame was derived from daily listing sheets that included walk-in females seeking general outpatient services. A systematic sampling method was applied to the list of eligible women who were seen in the primary care clinic during the study period between December 2014 and April 2015.

Women were eligible for the study if they were citizens of Saudi Arabia and aged 20 to 50 years. Women were excluded from the study if they were pregnant, lactating, or post-menopausal (defined as amenorrhea for at least six months). Self-reported disease conditions that may influence vitamin D metabolism, including renal diseases, liver diseases, hyperthyroidism or hypothyroidism, metabolic bone disease, malabsorption, cancer, diabetes, or women taking medications known to affect bone metabolism, were also excluded from the study. All women gave their written informed consent prior to participating in the study.

### Data collection

#### Demographic variables

Sociodemographic variables included age (years), marital status, e.g., married (living with partner) or unmarried (single, divorced/widowed); Occupation status (employed, unemployed, student, housewife); Monthly household income was divided into categories, according to the Central Department of Statistics and Information (2006/2007) and a previous published study [27], depending on the amount of Saudi Arabian Riyal (1SAR = 0.266USD): (< 4,000; 4,000 to 7,999; 8,000 to 14,999; 15,000 to 24,999; ≥ 25,000); residence (rented apartment, owned apartment, rented house, owned house, others) Participants were asked about the number of years of formal education, and then level of education was categorized as follows: (less than college, college graduate, and post graduate).

#### Dietary vitamin D intake

Dietary intake of vitamin D per day was estimated using the Semi-quantitative Food-Frequency Questionnaire (SFFQ). The SFFQ used was a modified version of a previously validated questionnaire [28], which was also validated in United Arab Emirates and showed positive correlation between the SFFQ and 3-day diet food records was (*r* = 0.74, *p* < 0.001) for vitamin D [29]. Vitamin D values of some food items were obtained from the Arabian Gulf Food Composition Table [30] such as meat, and local fish consumed in this area. However, because vitamin D values for non-local foods such as tuna and salmon are not available in the Arabian Gulf Food Composition Table, the U.S. Department of Agriculture reference data [31] were used. Vitamin D values of supplements and local food items such as vitamin D fortified milk and Laban drink (buttermilk) were obtained from food labels. Supplemental vitamin D and multivitamins were assessed by the SFFQ.

The selection of food items in SFFQ was obtained and reviewed with the investigator using visual estimates (photographs) to help subjects estimate average portion size. On the SFFQ, respondents were asked to record how often they consumed a single serving of each food listed during the previous month, with possible responses ranging from less than once per month to 2 or more per day. Vitamin D intake was computed by multiplying the intake frequency by the nutrient content of the portion size of specific items. Intake of vitamin D was calculated by summing nutrient intake from diet and supplemental sources. To determine the adequacy of vitamin D, the value obtained was assessed using Estimated Average Requirements (EAR) of 400 IU/day [4].

#### Sun exposure and skin type

The sun exposure recall questionnaire included details of the time of the day of the sunlight exposure; length of exposure to sunlight during the previous month on week days and weekends. Cover status when they were outside in the public area was used to determine the area exposed to direct sunlight. Women were classified as either (1) face covered but hands exposed, (2) face and hands exposed. In addition, women were asked the average length time per day they spent in their courtyard. In Saudi culture this is the place where the sunlight exposure occurs because in all other outdoor activities women generally cover their bodies by wearing black abayas [32].

Skin color was assessed by the investigator using the validated Fitzpatrick skin-type scale [33]. The system is a numerical classification scheme for determining six different skin types based on a questionnaire related to an individual’s genetic constitution, reactions, vulnerability to sunlight or UVB radiation, and tanning habits [33, 34]. The response to each question was measured on a scale of 0–4. The responses to all questions were summed to obtain the total score corresponding to the Fitzpatrick skin-type scale, which is as follows [33]: (I) pale white skin that always burns and never tans; (II) fair skin that burns easily and sometimes tans; (III) fair to medium skin that moderately burns and always tans gradually; (IV) medium skin that burns minimally and tans easily; (V) medium skin that rarely burns and always tans darkly; and (VI) olive to dark skin that never burns and tans darkly.The Fitzpatrick skin color scale has been previously used in Arabian Gulf studies including, United Arab Emirates [35] and Kuwait [36].

#### Knowledge of vitamin D and attitudes and behavior toward sun exposure

The content and face validity of the questions were ensured by reviewing relevant literature and questionnaires on vitamin D research and consulting with experts in the field. In addition, the questionnaire was pre-tested in pilot interviews in a similar setting with 30 volunteer women of the same age groups. The internal consistency reliability of the vitamin D knowledge test was calculated on the dichotomized items (correct/incorrect) using kuder-Richardson Formula 20. The reliability of the test was calculated to be 0.70, indicating that the test demonstrated acceptable reliability [37].

The questionnaire consisted of two sections. In the first section, the participants were asked seven multiple-choice questions about their knowledge of the sources and role of vitamin D. The questions were adapted from those used in previous validated surveys [21, 22] and were modified for our study population. The first question was used to screen participants. Women were asked if they had heard of vitamin D, and only women who had heard of vitamin D were asked to complete the rest of the survey. Questions 2–7 were used to calculate participants’ total knowledge score by adding the points received for each answer. One point was assigned for each correct answer, and 0 points were given for each incorrect answer or “I don’t know” response. If a question had two correct answers, then each correct answer was worth 0.50 points. If a question had three correct answers, then each correct answer was worth 0.33 points. The participants were regarded as more knowledgeable if they correctly answered three or more questions. The participants who answered question 1 negatively or correctly answered fewer than three questions were considered to be less knowledgeable about vitamin D.

The second section of the questionnaire included eight multiple-choice statements about women’s attitudes and behavior toward sunlight exposure. The statements were adapted from those used in a previous survey [38] and modified for our study population. This section included questions on whether women spend time in the sun or tanning, use sunscreen, wear a colored *abayas* during sun exposure, avoided sun exposure, and their reasons why.

#### Biochemical analysis

Non-fasting venous blood samples were drawn for serum 25-hydroxyvitamin D 25(OH)D. Quantitative 25(OH)D in serum was determined by using one step delayed chemiluminescent microparticle immunoassay (CMIA) (Architect, Abbott, Germany). Intra-assay 25(OH) D coefficient of variation (CV) from daily quality control was 1.4- 3.7% and inter-assay (CV) was 2.6– 4.6%. The fully automated Architect assay is designed to have an imprecision of ≤ 10%.

The serum samples were taken in the laboratory in the primary care clinic; after blood collections, samples were left for 10 min for clotting then immediately centrifuged at 3000 rpm for 10 min. Then, samples were aliquoted and frozen at -20° C until further analysis. Samples were run simultaneously in a batch. All analyses were performed in the Pathology laboratory at King Abdul-Aziz Medical City. Baseline serum 25(OH)D levels were classified into three categories according to the Institute of Medicine and WHO [4, 39] severe vitamin D deficiency serum 25(OH)D < 25 nmol/L, deficiency serum 25(OH)D 25–< 50 nmol/L, and sufficiency serum 25(OH)D ≥ 50 nmol/L.

### Statistical analysis

Data were analyzed using the Statistical Package for Social Science (SPSS) software version 22.0 (SPSS, Inc., Chicago, IL, USA). The characteristics of the study population were described through frequencies and percentages for categorical variables and summary statistics for continuous variables.

Vitamin D knowledge scores were dichotomized into two groups: less knowledgeable vs. more knowledgeable. Binary logistic regression was used to determine if there was an association between vitamin D knowledge level and, marital status, employment status, income level, age and education groups. Chi-square tests of independence were used to determine if there was an association between any items in vitamin D knowledge questionnaire and age of the participants. For skewed variables (serum vitamin D, vitamin D intake, fish intake, length of sun exposure), differences between the two groups (vitamin knowledge groups) were tested using Mann–Whitney U test.

Multiple linear regressions were conducted to determine the relationship between (1) total vitamin D intake and (2) serum 25(OH)D level and knowledge of vitamin D, before and after controlling for age and years of education. Ordinal logistic regressions were conducted to investigate if there was a relationship between attitudes and behaviors toward sun exposure, and serum vitamin D level (a 3-level categorical variable: severe deficiency, deficiency and sufficiency). Age and education levels were included as control variables. After log transformed serum 25(OH)D and length of sun exposure, general linear models were conducted to investigate if there was an relationship between (1) serum 25(OH)D and attitude toward sun exposure, after controlling for age and total vitamin D intake, (2) length of sun exposure and attitude toward sun exposure, after controlling for age and education level, (3) serum 25(OH)D and wearing colored abaya, after controlling for age and total vitamin D intake, and (4) length of sun exposure and wearing colored abaya, after controlling for age and education level. Bonferroni’s method was applied where applicable for the results of pairwise comparisons. For any test, a *p* value less than 0.05 indicated significance.

## Results

### Characteristics of the study population

A total of 257 premenopausal Saudi women aged 20–50 years from Jeddah were enrolled in this study between December 2014 and April 2015. Of these, 250 women completed bloodwork to test their serum 25(OH)D levels, whereas 7 women who did not complete bloodwork were excluded from the analysis of serum 25(OH)D levels. The mean age was 29.8 ± 7.4 years, and 59.5% of the participants were within the younger age group (20–30 years old). The median vitamin D intake from diets and supplements was 236.4 IU/day. More than half of the women (65%) were below the EAR for vitamin D (400 IU/day). Thirty seven percent of women reported using vitamin D supplements or multivitamins containing vitamin D in the past 30 days. The median duration of sunlight exposure was 9.4 min/day, and 22.6% women reported daily exposure to sun more than 30 min/day between 10 am to 4 pm, and 77.7% women reported exposure to sun less than 30 min/day. Further population characteristics are shown in Table 1. Among the 250 women who had a blood sample, the median concentration of 25(OH)D was 34.2 nmol/L. There was a high proportion of participants (77.6%) with vitamin D deficiency (25(OH)D < 50 nmol/L) (Table 1). Among those with the deficiency, 52 women were severely deficient (25(OH)D < 25 nmol/L) (52/250, 20.2% of the entire sample).

**Table 1:** Sociodemographic and lifestyle characteristics of Saudi premenopausal women (20–50 years) attending the primary health care center at KAMC, Jeddah (*n* = 257)

Characteristics	*n* (%)
Age (years)	
20–30	153 (59.5)
31–40	73 (28.4)
41–50	31 (12.1)
Education status	
Less than college	74 (28.8)
College graduate	163(63.4)
More than College	20 (7.8)
Income level (SR per month)	
< 4000	15 (5.8)
4000–< 8000	50 (19.5)
8000–< 15,000	78 (30.4)
15,000–< 25,000	53 (20.6)
≥ 25,000	36 (14.0)
Occupation	
Employed	133 (51.8)
Unemployed	124 (48.2)
Type of residence	
Rented (apartment/home)	114 (44.4)
Owned (apartment/home)	143 (55.6)
Marital status	
Unmarried (single/separated/divorced)	145 (56.4)
Married	112 (43.6)
Daily sun exposure (min/day)	
< 30 min/day	199 (77.4)
≥ 30 min/day	58 (22.6)
Area of skin exposed	
Face covered but hands exposed	164 (63.8)
Face and hands exposed	93 (36.2)
Skin color type	
II	6 (2.3)
III	106 (41.2)
IV	132 (51.4)
V	13 (5.1)
Vitamin D intake (IU/day) diet + supplement	
< 400	167 (65.0)
≥ 400	90 (35.0)
Vitamin D intake of supplement users	
< 400	13 (13.5)
≥ 400	83 (86.5)
Vitamin D supplement use (IU/day)	
No	161 (62.6)
Yes	96 (37.4)
Serum 25(OH)D level (nmol/L)	
Deficiency < 50 nmol/L	194 (77.6)
Sufficiency ≥ 50 nmol/L	56 (22.4)
Serum 25(OH)D level (nmol/L) of supplement users	
Deficiency < 50 nmol/L	47 (49.0)
Sufficiency ≥ 50 nmol/L	49 (51.0)

Abbreviations: Frequency (*n*); Percentage (%)

### Knowledge of vitamin D

Knowledge of vitamin D for the participants is summarized in Table 2. Of the 257 participants, 99% have heard of vitamin D, and 97% of participants thought vitamin D was important for their health. Sunlight was correctly identified as the single most important source of vitamin D by 91% of the participants. Only 37% of the participants have heard about vitamin D fortification in food. The top two dietary sources for vitamin D correctly identified by the participants were oily fish (48%) and fortified dairy products (36%). Only 5% thought meat was source of vitamin D, while 33% of participants indicated incorrect vitamin D sources such as leafy vegetables and fruits. According to the participants, the most important effects of vitamin D were osteoporosis prevention (75.7%) and bone health (72.9%). Less than half 48% of the participants identified incorrect health effects of vitamin D such as vision health, and skin softness. Nearly 80% of participants believed that avoiding sun exposure would decrease the amount of vitamin D an individual can get followed by sun screen use (25.9%) and older age (22.7%), and approximately 9% indicated an incorrect response such as fatty diets.

**Table 2:** Knowledge of vitamin D among Saudi premenopausal women aged 20–50 years attending the primary health care center at KAMC Jeddah (*n* = 257)

Knowledge of vitamin D	*n* (%)
Having heard/learnt about vitamin D	
Yes	255 (99.2)
No	2 (0.8)
Vitamin D is important for health	
Yes	248 (96.5)
Don’t know	7 (2.7)
Most important source of vitamin D	
Natural food sources	6 (2.3)
Sunlight**	233 (90.7)
Supplements	4 (1.6)
Do not know	12 (4.7)
Heard about vitamin D fortification in food	
Yes	95 (37.0)
No	160 (62.3)
Dietary sources for vitamin D^a^	
Oily fish**	122 (47.5)
Fortified dairy products**	92 (35.8)
Leafy vegetables	65 (25.5)
Eggs**	64 (25.1)
Citrus fruits	19 (7.5)
Meat**	12 (4.7)
Most important effect of vitamin D^a^	
Bone health**	186 (72.9)
Osteoporosis prevention**	193 (75.7)
Hair growth	87 (34.1)
Immunity**	51 (20.0)
Skin softness	21 (8.2)
Vision health	14 (5.5)
Factors that can decrease the amount of vitamin D^a^	
Avoid of sun exposure**	199 (78.0)
Sunscreen**	66 (25.9)
Age**	58 (22.7)
Skin color**	33 (12.9)
Fatty diet	24 (9.4)

Participants were able to choose more than one option therefore percentages do not add up to 100%

Abbreviations: Frequency (n); Percentage (%)

** Indicate the correct answer

### Association between knowledge of Vitamin D and sociodemographic factors

The average score of knowledge of vitamin D was 3.40 ± 1.1 with a minimum score of 0 and a maximum score of 5.75. Approximately 63% participants were more knowledgeable on vitamin D compared to 37% who were less knowledgeable on vitamin D.

Table 3 shows the results of the binary logistic regression indicated that there was a statistically significant association between vitamin D knowledge level and marital status (*χ*^2^(1) = 4.121, *p* = 0.040) and education (*χ*^2^(2) = 7.886, *p* = 0.019). In particular, participants who were married were less likely to be have higher level of vitamin D knowledge than participants who were unmarried (OR = 0.522, 95% CI = (0.281, 0.971)). Furthermore, participants who had less than college education were less likely to be have higher level of vitamin D knowledge than participants were college graduates (OR = 0.397, 95% CI = (0.206, 0.765)). No significant association were detected between vitamin D knowledge level and other sociodemographic variables, including employment status, income level, and age. However, some items in vitamin D knowledge questionnaire differed significantly among age groups. Younger participants were more aware of sunlight as source of vitamin D than older participants (*χ*^2^(2, 255) = 10.097, *p* = 0.007, Additional file 1: Figure S1). Conversely, older participants were more aware of oily fish as source of vitamin D (*p* = 0.02, Additional file 1: Figure S2).

**Table 3:** Associations between vitamin D knowledge score and demographics variables among Saudi premenopausal women aged 20–50 years attending the primary health care center at KAMC Jeddah (*n* = 232)

Variable	N	Wald chi-square statistic (df)	*p*	Odds ratio (95% CI)
Age		2.505 (2)	0.286	
20–30	136			Ref
31–40	66			1.162 (0.568, 2.376)
41–50	30			2.154 (0.832, 5.577)
Marital status		4.212 (1)	0.040*	
Unmarried (single/separated/divorced)	124			Ref
Married	108			0.522 (0.281, 0.971)
Education status		7.886 (2)	0.019*	
College graduate	144			Ref
Less than college	71			0.397 (0.206, 0.765)
More than college	17			0.636 (0.214, 1.891)
Employment status		0.933 (1)	0.334	
Unemployed	115			Ref
Employed	117			1.380 (0.718, 2.655)
Income level (SR per month)		0.060 (1)	0.806	
< 15,000	143			Ref
≥ 15,000	89			1.081 (0.582, 2.008)

The logistic regression was modeling the probability of "more knowledgeable”

Ref = reference category

*indicates significance at the 0.05 level

### Association between knowledge of vitamin D and vitamin D intake

**Fig. 1:**
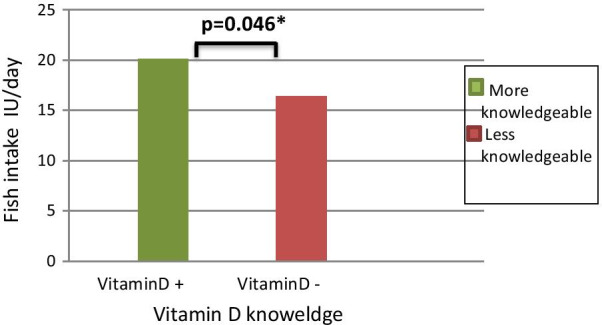
Median fish intake IU/day by vitamin D knowledge groups. *indicates significance at the 0.05 level

In the bivariate analysis, the association between vitamin D knowledge level and fish intake is shown in Fig. 1. Median fish intake in the vitamin D knowledgeable group (20.1 IU/day) was significantly higher compared to vitamin D intake in the less knowledgeable group (16.4 IU/day) (*p* = 0.046). The association remained significant after adjustment for energy intake or after exclusion of women that used vitamin D supplementation. No significant difference was observed between vitamin D knowledge level groups and other vitamin D intake sources.

The results of the multiple linear regression models with log transformed total vitamin D intake as the dependent variable and knowledge of vitamin D score as the independent variable (Additional file 1: Table S1) suggested that there was no statistically significant relationship between total vitamin D intake and knowledge of vitamin D, before and after controlling for age and years of education (before controlling for age and education: t(255) = 0.510, *p* = 0.610; after controlling for age and education: t(253) = 0.343, *p* = 0.732).

### Association between knowledge of vitamin D and serum 25(OH)D level

The results of multiple linear regression model with log transformed serum 25(OH)D level as the dependent variable and knowledge of vitamin D score as the independent variable (Additional file 1: Table S2) suggested that there was no statistically significant relationship between serum vitamin D level and knowledge of vitamin D, before and after controlling for age and years of education (before controlling for age and education: *t*(248) = 1.546, *p* = 0.123; after controlling for age and education: *t*(246) = 0.913, *p* = 0.362).

### Attitudes and behavior toward sunlight exposure and serum vitamin D levels

Responses from 257 women who answered questions regarding sun exposure are summarized in Additional file 1: Table S3. 57.6% of women believed that sunlight exposure is good and healthy. Half of the participants 50% rarely like going in the sun, and about 45% did not use sun screen products. The majority of women 70% never wore colored abaya (wore the traditional black abaya) when they went outside in the sun. Natural and artificial tanning was not common practice among our participants; about 63% reported never exposed to the sun for this intension and 93% reported never seeked a suntan from artificial tan such as tanning bed. The main reasons for avoiding sun were prevent skin pigmentation and specific health reasons such as headache and hot weather.

**Fig. 2:**
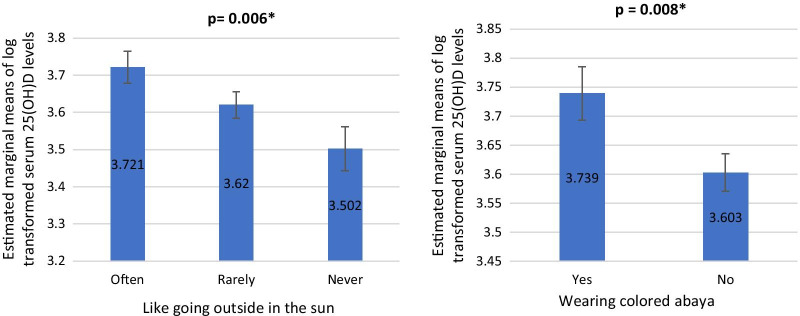
Serum 25(OH)D levels by attitude toward sun Exposure. Left: serum 25(OH)D levels by like going outside in the sun. Right: serum 25(OH) by wearing colored abaya *indicates significance at less than 0.05 level

There was no statistically significant association between attitudes and behaviors concerning sunlight exposure and serum vitamin D levels. However, there was a statistically significant difference in log (serum 25(OH)D levels) based on the attitude of sunlight exposure (like going outside in the sun), after controlling for age and total vitamin D intake (*F*(2, 243) = 5.188, *p* = 0.006). In particular, women who often went out outside in the sun had statistically significantly higher serum 25(OH)D levels than those who never (Fig. 2). There was also a statistically significant difference in log (serum 25(OH)D levels) based on the attitude of sunlight exposure (wearing colored abaya), after controlling for age and total vitamin D intake (*F*(1, 245) = 7.192, *p* = 0.008). In particular, women who wore colored abaya had statistically significantly higher serum 25(OH)D levels than those who did not (wore the traditional black abaya) (Fig. 2).

**Fig. 3:**
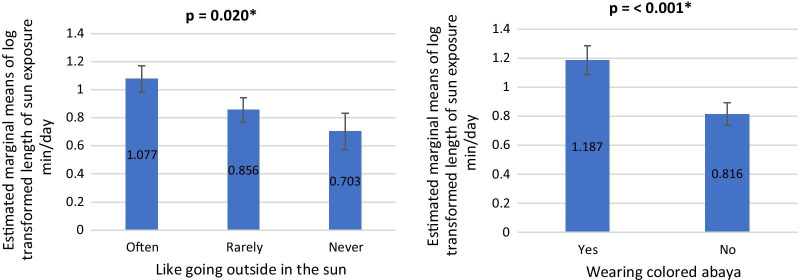
Length of sun exposure min/day by attitude toward sun exposure. Left: length of sun exposure min/day by like going outside in the sun. Right: length of sun exposure min/day by wearing colored abaya *indicates significance at less than 0.05 level

There was a statistically significant difference in log (length of sun exposure min/day) based on the attitude of sunlight exposure (like going outside in the sun), after controlling for age and education level (*F*(2, 249) = 3.951, *p* = 0.020). In particular, women who often went out outside in the sun had statistically significantly higher length of sun exposure min/day than those who never (Fig. 3). Finally, there was also a statistically significant difference in log (length of sun exposure min/day) based on the attitude of sunlight exposure (wearing colored abaya), after controlling for age and education level (*F*(1, 251) = 13.215, *p* < 0.001). In particular, women who wore colored abaya had statistically significantly higher length of sun exposure min/day than those who did not (Fig. 3).

## Discussion

This study provides insight regarding the current knowledge of the importance of vitamin D and attitudes toward sun exposure among premenopausal women attending the primary care clinic in Jeddah. Overall, it seems that women in Jeddah are more aware of vitamin D than those reported in the published literature [22, 40, 41]; for example, almost 99% of the women in the current study had heard of vitamin D, compared to 90% of parents of children (*n* = 1752) in Jeddah [42] and 63% of the men and women (*n* = 503, age 20–40 years old) in Sharjah, United Arab Emirates [40]. In comparison, one internationally published report stated that 73% of Chinese middle aged and elderly men and women (*n* = 547) had heard about vitamin D [22]. Moreover, a higher proportion of our participants identified the sun as the most important source of vitamin D, when compared to previous studies conducted in Sharjah [40], Hong Kong [22], Australia [41], and India [43] (91% vs. 43%, 23%, 83%, and 53.3%, respectively). Gaps in the basic knowledge about vitamin D have been observed in previous studies among Saudi school girls [44], female Saudi university students [25], and Saudi women [26]. The differences observed between the present study and previous studies may reflect the medical information about vitamin D provided to our participants while attending the primary health center, which may not be available to the general population.

In contrast, we observed a lack of knowledge when the women in this study were asked about vitamin D fortification or vitamin D intake sources, and less than half of the women were able to identify oily fish and fortified dairy products as sources of vitamin D. A minority of the women could name meat as a source of vitamin D, while about one-third of the women identified incorrect food sources of vitamin D, such as vegetables and fruits. These findings confirm the results of previous reports [19, 22, 25, 45, 46]. In a study conducted with Canadian university students (*n* = 1088), Boland et al. [46] underscored that university students have poor knowledge of vitamin D, which is particularly concerning because the food sources of vitamin D that were least identified by participants are those upon which Canadians must rely during their limited winter sun exposure due to the country’s high latitude. In the present study, the lack of knowledge about vitamin D food sources is also troubling among our participants who rely more on vitamin D intake sources than sun exposure. This is largely due to the covering of the body by traditional clothing (black *abaya*) and time spent indoors, which limits their sun exposure. Overall, these findings highlight the need for increased awareness and improved knowledge of vitamin D among women, particularly vitamin D intake sources, in future intervention programs.

Although more than two-thirds of the women in the current study were aware of the major benefits of vitamin D on bone health, their actual vitamin D dietary intake was only 236.4 IU/day, which is lower than the U.S. Estimated Average Requirement (EAR) of 400 IU/day. Limited knowledge regarding vitamin D sources could be one of the factors associated with the insufficient intake of vitamin D, and this relationship has been identified previously [46].

In the present study, we did not find a significant association between the knowledge score and age. However, we found that women 20–30 years old and 31–40 years old were more aware of sunlight as a main source of vitamin D than older women (41–50 years old), but they were not exposing themselves to the sun more often than the older women. This is consistent with research done in Hong Kong, in which Kung et al. [22] found that older women were less aware of vitamin D than women of a younger age, but they were more likely to seek sun exposure when compared to younger women. Conversely, with regard to food sources of vitamin D, we found that middle aged women (31–50 years old) were more aware of oily fish as a source of vitamin D when compared to younger women (20–30 years old). This awareness has been associated with certain behaviors among middle aged women, who tended to eat more fish when compared to younger women. In addition, the results of the present study indicated that women aged 20–30 years were the least knowledgeable about the benefits of vitamin D for bone health. This may be the result of the many Saudi public health education programs about the bone health benefits of vitamin D and calcium that have targeted middle-aged women.

The direct association between the educational level and vitamin D knowledge in the current study is not surprising, since previous studies have also found positive associations between knowledge and educational levels [40, 47]. Our results were especially concerning, because so many of the participants were highly educated women (college graduate), which suggests that the general population in Jeddah may be even less aware of vitamin D than the participants in the current study. In addition, unmarried women having better vitamin D knowledge than married women may be due to the higher education level of the former, which can result in their being better informed by their peer groups and receiving more information on vitamin D through their classwork. These findings suggest that vitamin D educational programs should target specific population groups (i.e., low education, married women) with the greatest need for information about vitamin D to implement any lifestyle changes [47].

In this study, we observed insufficient vitamin D intake among all women, regardless of their knowledge about vitamin D. The median dietary vitamin D intake for both groups was below the EAR of 400 IU/day. However, being aware that fish is a rich source of vitamin D was associated with an increase in fish consumption among our sample; the median fish intake in the vitamin D knowledgeable group was significantly higher than that in the less knowledgeable group. Although dietary sources alone do not provide sufficient vitamin D, dietary recommendations should be included in public health education programs to help reduce vitamin D deficiency, particularly among women with limited sun exposure [48]. Undoubtedly, improving knowledge about vitamin D in Saudi Arabia can motivate young women to adopt healthy eating behaviors and may be an effective step toward increasing women’s responsibility for preventing vitamin D deficiency and bone disease over the long term [22].

Overall, low serum 25(OH)D was observed in the present study, with 77.6% of the women having vitamin D deficiency (25(OH)D < 50 nmol/l). This deficiency was similar in women with more and less knowledge about vitamin D. This finding is inconsistent with a previous study conducted in the Netherlands by Oudshoorn et al. [21], which reported that participants with the most knowledge about vitamin D had a mean serum 25(OH)D level of 58.5 nmol/l, a level approximately twice that of participants with the least knowledge. In this study, the lack of association may be attributable to the high prevalence of vitamin D deficiency in our sample.

In the present study, the duration of sunlight exposure was low, and more than half of the women were either never exposed to the sun or exposed less than 10 min per day. These results are consistent with the findings of other studies conducted in Saudi Arabia [26] and Kuwait [49]. Moreover, a lack of consistency between the attitudes toward sun exposure and knowledge of vitamin D was found in this study. While the majority of the women believed that sun exposure was good for their health, and that the human body can obtain vitamin D through exposure to sunlight, more than half of the women disliked being exposed to the sun.

Concerns about the strength of the sun in Jeddah, symptoms such as fatigue and headache, a desire to prevent darkening of the skin, cultural norms requiring women to cover their bodies, and hot climatic conditions were the most common reasons for avoiding the sun. Despite being allowed only limited sun exposure in public areas for cultural reasons, many of the participants reported that they had private courtyards in their houses where they could spend some time in the sun without veils. Unfortunately, most of the women who did have courtyards in their houses tended to avoid direct exposure to sunlight because of the heat or for cosmetic reasons (e.g. minimizing skin discoloration and laser and chemical peel treatments).

However, concern about skin cancer risk was not the main reason for sun avoidance in the current study, which is dissimilar to the findings of previous studies in New Zealand [38, 50]. Moreover, lifestyles and personal choices were also identified as reasons to avoid the sun. More than half of our participants were employed, and they reported their long working hours indoors as the reason for not getting enough sun.

In the current study, there was a lack of information and clear advice regarding the amount of time that the women should be exposed to ultraviolet B radiation (UVB), and about what time of day an individual could obtain adequate sun exposure in order to improve their serum 25(OH)D levels. Therefore, the optimum sun exposure times during the summer in western region (Makkah) in Saudi Arabia was determined based on provitamin D3 conversion [51]. The study suggested that the optimum sun exposure times for vitamin D3 production in the western region during the summer months were from 8:30 am to 10:30 am and 2:00 pm to 4:00 pm, while the maximum UVB hours were between 10:30 am and 2:00 pm. These hours are believed to be most responsible for sun burns and skin cancer [51].

Healthcare professionals should be informed and updated about the guidelines for sun exposure in order to educate the general community, which may effectively help the general public increase their vitamin D status and prevent vitamin D deficiency throughout the country. A recent study in Saudi Arabia [52] suggested increasing the dietary intake of vitamin D (food and supplements) during the summer, because of the reduced amount of outdoor activity and extreme heat. However, Algamdi et al. [53] reported that 66% of Saudis spend extra time outdoors during the winter, when compared to the summer; therefore, sunlight exposure is a cost free and relatively risk-free option for restoring serum 25(OH)D levels [54].

A high proportion of our sample displayed uncertainty about whether or not sunscreen affects serum vitamin D levels. Only 26% of the women studied believed that the use of sunscreen could actually affect these levels. It should be noted that based on evidence from observational and experimental studies, we considered sunscreen use and older age as correct answers to the knowledge question about factors that can decrease serum 25(OH)D levels [2, 54]. However, no randomized controlled trials have reported that daily sunscreen use has a significant role in suppressing cutaneous synthesis of vitamin D [55]. Likewise, there is not much evidence that serum 25(OH)D levels lower with age. While previously published data suggest that vitamin D deficiency is more prevalent among older adults [2, 56], other study has found that mean serum 25(OH)D levels do not differ by age group in data from the National Health and Nutrition Examination Survey (NHANES) [57].

Darker skin pigmentation is also an effective sunscreen, and reduces the capacity of the skin to synthesize vitamin D from sunlight [2]. It has been suggested that 2–6 min of sun exposure per day in the summer, and 4–17 min per day in the winter are sufficient for vitamin D production in fair skinned individuals [41]. However, a high proportion of our sample was unaware of the effects of skin color on vitamin D production. Further emphasis is needed in public education regarding this issue, since the majority of our participants have skin types 3 and 4, and require longer sun exposure times than fair skinned individuals (skin types 1 and 2).

Protective traditional clothing, rather than sunscreen, was identified as the most commonly used sun protection measure in Saudi Arabia [53]. Saudi women usually wear traditional black *abaya* over their clothing, covering the whole body, with the exception of the hands and face (in some women), which are exposed to sunlight. In the present study, no differences were found in the serum 25(OH)D levels between those women who were fully veiled (covered their faces, but their hands were exposed) and partially veiled (exposed their faces and hands). Recently, some young Saudi women have begun wearing colored *abayas* (e.g., off-white, beige, gray, blue) particularly in Jeddah. In the current study, about 7% of the women usually wore colored *abayas*, and 23% of the women wore colored *abayas* only occasionally. The women who wore colored *abayas* in the sun had significantly higher serum 25(OH)D levels when compared to those women who never wore colored *abayas*.

Experimental biology studies have shown that clothing inhibits the production of previtamin D and reduces serum 25(OH)D levels, and that the transmission of previtamin D effective radiation depends on the fabric type [58, 59]. Salih [59] investigated the influence of different fabric samples used in Oman by both men and women on the photo transformation of 7-dehydrocholesterol to previtamin D3. The investigator found that the type of fabric used may greatly inhibit the photoproduction of vitamin D3. With regard to the color of the fabric, the investigator found that white fabric attenuates more light than other colors. However, Parisi et al. (2005) found that the effect of color was more related to the fabric type, with higher levels of synthesized of previtamin D under white than black eyelet fabric, while the reverse was observed for jersey fabric [60].

To the best of our knowledge, this is the first study to examine the relationships between vitamin D knowledge, attitudes, and practices toward sun exposure, and serum 25(OH)D levels among healthy premenopausal women living in Jeddah city. Our data provides important information for building appropriate intervention strategies for the prevention of vitamin D deficiency among young women in Jeddah. However, a few limitations have been identified; for example, the sample was derived from the primary health center at King Abdul-Aziz Medical City in Jeddah, and therefore, may not be generalizable to all women residing in Jeddah or in the other regions of Saudi Arabia. Furthermore, response bias may influence the results due to the high educational levels of our participants, which may account for the greater knowledge of vitamin D than the general population. Sun exposure practices could also be more common in this group than in the general population, since educational level has been linked to sun exposure [6, 25].

## Conclusion

A lack of knowledge about vitamin D sources and other factors affecting vitamin D status remains high in this sample of premenopausal women in Jeddah. Moreover, there is a conflict between information regarding the benefits of sunlight as a main source of vitamin D and negative attitudes toward sun exposure among many young women who are at the greatest risk of vitamin D deficiency. Educational programs should place more emphasis on improving knowledge of dietary sources of vitamin D and the importance of sunlight and should provide information concerning the best time of day for obtaining adequate sun exposure, particularly during winter. We also recommend an increase in the availability of women-only areas in cities for those who do not have their own private space by designating areas in neighborhood parks and workplaces, such as private gardens, for women to take in sunlight. Further studies considering the magnitude of sunlight attenuation by *abayas* of different colors and fabrics may be needed to determine whether specific colors or fabrics will directly affect the photosynthesis of vitamin D.

## Supplementary Information

**Additional file 1.** Knowledge and Attitudes about vitamin D and Sunlight Exposure in Premenopausal Women Living in Jeddah, and their Relationship with Serum Vitamin D Levels.

## Data Availability

The data supporting the conclusions of this article are included within the manuscript. The dataset could be obtained from the corresponding author upon reasonable request.

## Abbreviations

25(OH)D: 25-Hydroxyvitamin D
KAMC: King Abdul-Aziz Medical City
CMIA: Chemiluminescent microparticle immunoassay
CI: Confidence interval
OR: Odds ratio
SFFQ: Semi-quantitative Food-Frequency Questionnaire
EAR: Estimated Average Requirements
UVB: Ultraviolet B radiation
IU: International unit
SAR: Saudi Arabian Riyal
USD: United States Dollar
