# Supplementary material for: Knowledge and attitudes about vitamin D and sunlight exposure in premenopausal women living in Jeddah, and their relationship with serum vitamin D levels
Source: J Health Popul Nutr. 2021 Aug 28;40:38. doi: 10.1186/s41043-021-00263-w (PMC8403372; doi:10.1186/s41043-021-00263-w)
Supplement: Supplementary file 1 — Additional file 1. Knowledge and Attitudes about vitamin D and Sunlight Exposure in Premenopausal Women Living in Jeddah, and their Relationship with Serum Vitamin D Levels. [file 41043_2021_263_MOESM1_ESM.docx]

# Knowledge and Attitudes about vitamin D and Sunlight Exposure in Premenopausal Women Living in Jeddah, and their Relationship with Serum Vitamin D Levels

Tahani A. Zareef,^1,2,3^ Robert T. Jackson^1^

**FIGURE 1S.** Percentage of women aware of sunlight as main source of vitamin D stratified by age.

* indicates significance at less than 0.05 level.

**FIGURE 2S.** Percentage of women aware of oily fish as source of vitamin D stratified by age.

* indicates significance at less than 0.05 level.

**Supplementary Table S1:** Association between Knowledge of Vitamin D and Vitamin D Intake

for Saudi premenopausal women attending the primary health care center at KAMC, Jeddah

|  | **B** | **SE** | **t** | **p-value** |  |
| --- | --- | --- | --- | --- | --- |
| **Model 1**  Vitamin D knoweldge score | 0.016 | 0.032 | 0.510 | 0.610 |  |
| **Model 2** |  |  |  |  |  |
| Vitamin D knoweldge score | 0.011 | 0.033 | 0.343 | 0.732 |  |
| Age | 0.018 | 0.005 | 3.932 | < 0.001* |  |
| Years of education | -0.001 | 0.011 | -0.045 | 0.964 |  |

*Note: Dependent variable = vitamin D intake . n = 257. B = Unstandardized regression coefficients. SE = standard error. t = t-statistic. p=p-value. * indicates significance at the 0.05 level. Model1 Unadjusted , Model 2 Adjusted for aage and years of education*

**Supplementary Table S2:** Association between Knowledge of Vitamin D and Serum 25(OH) D Level for Saudi premenopausal women attending the primary health care center at KAMC, Jeddah

|  | B | | SE | t | p-value |  |
| --- | --- | --- | --- | --- | --- | --- |
| **Model 1**  Vitamin D knoweldge score | | 0.019 | 0.012 | 1.546 | 0.123 |  |
| **Model 2** | |  |  |  |  |  |
| Vitamin D knoweldge score | | 0.011 | 0.012 | 0.913 | 0.362 |  |
| Age | | 0.009 | 0.002 | 5.333 | < 0.001* |  |
| Years of education | | 0.006 | 0.004 | 1.442 | 0.151 |  |

*Note: Dependent variable = log transformed serum vitamin D level . n = 250. B Unstandardized regression coefficients. SE = standard error.* *t = t-statistic. p=p-value.* indicates significance at the 0.05 level. Model 1 Unadjusted , Model 2 Adjusted for age and years of education.*

**Supplementary Table S3:** Attitudes and behavior toward sunlight exposure among Saudi premenopausal women aged 20-50 years attending the primary health care center at KAMC Jeddah (n=257).

|  |  | |  | |  |
| --- | --- | --- | --- | --- | --- |
| **Sun exposure questions** | | **Responses** | | ***n(%)*** | |
| 1. I believe sunlight exposure is good for my health | | Always | | 148 (57.6) | |
|  | | Sometimes | | 108 (42.0) | |
|  | | Never | | 1 (0.4) | |
| 2. I like going outside in the sun | | Often | | 80 (31.1) | |
|  | | Rarely | | 129 (50.2) | |
|  | | Never | | 47 (18.3) | |
| 3. I enjoy spending time outside (courtyards) in the sun | | Often | | 25 ( 9.7) | |
|  | | Rarely | | 87 (33.9) | |
|  | | Never | | 46 (17.9) | |
| 4. I use sunscreen when I go outside | | Always | | 38 (14.8) | |
|  | | Sometimes | | 51 (19.8) | |
|  | | Rarely | | 53 ( 20.6) | |
|  | | Never | | 115 ( 44.7) | |
| 5. I wear colored abaya when I go outside in the sun | | Always | | 19 (7.4 ) | |
|  | | Sometimes | | 59 (23.0) | |
|  | | Never | | 179 (69.6) | |
| 6. I spend time in the sun (in private area) in order to get a tan | | Often | | 21 (8.2) | |
|  | | Rarely | | 75 (29.2) | |
|  | | Never | | 161 (62.6) | |
| 7. I seek a suntan from artificial tan such as tanning bed | | Often | | 1 (0.4) | |
|  | | Rarely | | 16 (6.2) | |
|  | | Never | | 239 (93.0) | |
| 8. The primary reason for avoiding the sun is | | Custom/religion  Public health messages | | 22 (8.6)  4 (1.6) | |
|  | | Specific health reasons | | 34 (13.2) | |
|  | | Hot weather | | 20 (7.8 ) | |
|  | | To avoid dark skin | | 101 (39.3) | |
|  | | Do not avoid the sun | | 108 (42.0) | |
| 9.I would spend more time in the sun if ^a^ | | I wasn’t worried about skin cancer | | 24 (9.3) | |
|  | | I had more time | | 106 (41.0) | |
|  | | I had more privacy | | 76 (29.6) | |
|  | | I would not spend more time in the sun | | 98 ( 38.1) | |

*Note: For “I like going outside in the sun” and “I seek a suntan from artificial tan such as tanning bed”, participants were excluded from the analysis if answering “Don’t know” (n = 255). For “I enjoy spending time outside (courtyards) in the sun”, participants were excluded from the analysis if answering “No courtyards” (n= 154). ^a^ Participants were able to choose more than one option therefore percentages do not add up to 100%. Abbreviations: Frequency (n); Percentage (%).*
